# Mobility outcomes and associated factors of acute geriatric care in hospitalized older patients: results from the PAGER study

**DOI:** 10.1007/s41999-023-00869-9

**Published:** 2023-10-01

**Authors:** Christian Werner, Laura Bauknecht, Patrick Heldmann, Saskia Hummel, Michaela Günther-Lange, Jürgen M. Bauer, Klaus Hauer

**Affiliations:** 1 Geriatric Center, Heidelberg University Hospital, Agaplesion Bethanien Hospital Heidelberg, Rohrbacher Str. 149, 69216 Heidelberg, Germany; 2 Medical Faculty Heidelberg, Heidelberg University, Im Neuenheimer Feld 672, 69120 Heidelberg, Germany; 3 Network Aging Research (NAR), Heidelberg University, Bergheimer Str. 20, 69115 Heidelberg, Germany; 4 Division of Physiotherapy, Department of Applied Health Sciences, Hochschule für Gesundheit (University of Applied Sciences), Gesundheitscampus 6-8, 44801 Bochum, Germany; 5 Department of Clinical Gerontology, Robert-Bosch-Hospital, Auerbachstraße 110, 70376 Stuttgart, Germany

**Keywords:** Hospitalization, Patient care, Geriatrics, Mobility limitation, Physical activity, Frailty

## Abstract

**Purpose:**

To examine distinct mobility outcomes (locomotor capacity, physical activity, life-space mobility) of acute geriatric care (AGC) in acutely hospitalized older adults and identify predictors associated with these outcomes.

**Methods:**

The PAGER study was designed as a prospective observational study**.** Mobility outcomes of 107 hospitalized older patients (age = 83.2 ± 6.4 years, female: *n* = 68, 63.6%) receiving AGC were measured at hospital admission and discharge. Locomotor capacity was assessed with the Short Physical Performance Battery (SPPB), 24-h physical activity (step count) with an activity monitor, and life-space mobility with the Life-Space Assessment in Institutionalized Settings (LSA-IS). Baseline demographical, clinical, physical, cognitive, and psychological characteristics were analyzed as candidate predictors of mobility outcomes.

**Results:**

SPPB (median [interquartile range] 4.0 [2.8–5.0] pt. vs. 5.0 [3.0–6.3] pt.), step count (516 [89–1806] steps vs. 1111 [228–3291] steps), and LSA-IS total score (10.5 [6.0–15.0] pt. vs. 16.3 [12.0–24.1] pt.) significantly improved during AGC (all *p* < 0.001). Adjusting for baseline status, frailty was identified as an independent negative predictor of SPPB, step count, and LSA-IS at discharge (*p* = 0.003–0.005). Barthel Index was also independently positively associated with step count (*p* = 0.017) at discharge, as was the mean daily PA level with SPPB (*p* = 0.027) at discharge, both independent of baseline status.

**Conclusion:**

AGC improves distinct mobility outcomes in hospitalized older patients. Frailty was consistently found to be an independent negative predictor of all mobility outcomes. Frailty assessment in AGC may be important to identify patients at risk for decreased treatment gains in mobility. Early PA promotion in AGC seems to be beneficial in improving patients’ locomotor capacity.

**Supplementary Information:**

The online version contains supplementary material available at 10.1007/s41999-023-00869-9.

## Key summary points

**Aim:**

To examine different mobility outcomes of acute geriatric care in acutely hospitalized older adults and identify associated factors.

**Findings:**

Patients showed significant increases in locomotor capacity, physical activity, and life-space mobility from hospital admission to discharge, for which frailty was consistently identified as a negative independent predictor. A higher mean daily physical activity level was independently predictive of improvements in locomotor capacity.

**Message:**

Older hospitalized patients benefit from acute geriatric care in terms of distinct mobility outcomes; however, frailty-specific adaptations may be needed for frail patients to optimize their mobility outcomes.

**Supplementary Information:**

The online version contains supplementary material available at 10.1007/s41999-023-00869-9.

## Introduction

Mobility can broadly be defined as an individual’s ability to move independently around in the environment and is crucial for autonomy, participation, and quality of life [1]. Older adults acutely admitted to hospital are at extraordinary risk of mobility decline due to high levels of physical inactivity. Low physical activity (PA), as measured by body-worn accelerometers, during hospitalization is common among these patients, spending 71 to 93% of the time lying in bed [2, 3] and showing very low levels of “on-feet” activity, with mean or median daily step counts between 478 and 846 steps [4–7] and mean or median daily standing and/or walking durations between 12 and 102 min per day [2, 3, 7, 8]. Direct observations and patient surveys also suggest that the spatial extent of mobility in the hospital is very restricted in older patients. It has been reported that they spent up to 90% of the day in their own room [9] and only 0.2 to 2.4% outside their own ward [9, 10], that only 19 to 27% of them walked in the hallway during daytime [11, 12], and that 48% do not leave their room at all throughout the hospital stay [13].

Physical inactivity can rapidly lead to a dramatic loss of muscle mass and strength in older adults [14], and several studies have shown that hospitalized older patients with low PA and/or restricted in-hospital mobility are at increased risk of numerous negative outcomes, such as hospital-acquired functional decline, longer hospital stay, hospital readmission, institutionalization, and mortality [4, 6, 15–17]. Despite being a highly relevant biomarker of functional recovery and trajectory, mobility seems to have long been a rare outcome of hospital care in older patients, not having been routinely assessed or specifically targeted for increasing during hospitalization [18].

Mobility assessments can provide distinct information on a patient’s mobility status. Laboratory-based assessments of specific mobility-related tasks (e.g., standing, walking, or getting up) refer to locomotor capacity as the highest possible level of physical functioning at a given moment in time [19]. In contrast, assessments of PA, defined as bodily movement in daily life that results in energy expenditure [20], and of life-space mobility (LSM), defined as the spatial extent, frequency, and independence of an individual’s movement in daily life [21], refer to how the potential (locomotor capacity) is realized in non-standardized, real-word environments over a longer period of times [19].

As a complement to the curative treatment of the medical condition, the goal of acute geriatric care (AGC) models is to maintain and enhance patient’s independence in activities of daily living (ADLs) and mobility, and to prevent hospital-acquired functional decline through early mobilization and rehabilitation [22, 23]. AGC typically includes a comprehensive geriatric assessment, multidimensional therapeutic strategies tailored to the complex and individual needs of patients, early physical rehabilitation, regular team meetings with all health professionals involved in care processes (i.e., geriatrician, nurse, occupational therapist, physiotherapist, psychologist, social workers), clinician leadership, prepared environment, patient-centered goal setting, and early discharge planning for transition of care [22–25]. In Germany, comprehensive AGC is delivered during an acute hospital stay and lasts between 7 and 21 days [26].

The benefits of AGC on ADL functioning of acutely hospitalized older patients have been widely documented [24, 25]. However, those with distinct mobility outcomes have not yet been examined in detail among this patient population. Some observational studies in older patients admitted to acute geriatric hospital wards reported an improvement in locomotor capacity (e.g., Δ Timed Up and Go =  + 27.3–28.1% [27], Δ Performance Oriented Mobility Assessment =  + 33–75% [28]) or an increase in PA (Δ median daily step count =  + 77–130%, Δ median standing and/or walking duration: + 120–150% [5, 8]) over hospital stay; however, findings on LSM outcomes of AGC are lacking.

Analyzing predictors of intervention outcomes is an important step towards identifying factors that may counteract treatment success, (further) developing tailored, patient-centered and effective therapeutic approaches, and optimally managing healthcare resources. Over the last decades, several patient characteristics predictive of functional outcomes of acute hospital care and/or geriatric inpatient rehabilitation have been identified, such as age, medication, comorbidities, nutritional, cognitive and functional status, frailty, depressive symptoms, fear of falling (FoF), locomotion mode, locomotor capacity, in-hospital mobility, and PA [13, 15–17, 29–34]. However, there is limited knowledge about predictors of mobility outcomes in hospitalized older patients. The few studies conducted in this patient population indicated that age, gender, cognitive impairment, underweight, sensory impairment, and/or frailty as personal factors are predictive of changes in locomotor capacity over hospital stay [35–37]; however, predictors of PA and LSM outcomes are still unknown.

Overall, the aim of this study was to examine distinct mobility outcomes (locomotor capacity, PA, and LSM) of AGC in hospitalized older patients and to identify predictors associated with these mobility outcomes.

## Methods

### Study design and setting

The PAGER (“Physical Activity in Geriatric patients during early Rehabilitation”) study was designed as a pragmatic, prospective observational cohort study to investigate longitudinal changes in distinct mobility outcomes from hospital admission to discharge in acutely hospitalized older patients receiving AGC. The study was conducted between January and August 2019 in a German geriatric hospital (Agaplesion Bethanien Hospital Heidelberg, Germany). The Ethics Committee of the Medical Faculty Heidelberg approved the study protocol (S-709/2018) in accordance with the principles of the Declaration of Helsinki, and all participants (and legal representatives) provided written informed consent to participate prior to study inclusion. The study was prospectively registered at the German Clinical Trials Register (DRKS00016028) on January 29, 2019.

### Participants

All patients consecutively admitted to the acute geriatric wards of the hospital during the 8-month study period were screened for eligibility. Inclusion criteria were receipt of AGC (so-called “early rehabilitative geriatric complex treatment”, OPS code 8-550) according to the German Operation and Procedure Classification System (OPS), which is the official classification system for the coding of operations, procedures and general medical measures in the German inpatient sector [26], age ≥ 65 years, ability to walk ≥ 4 m with or without walking aid, adequate German language skills, and written informed consent within 72 h after hospital admission. Exclusion criteria included severe cognitive impairment (Mini-Mental State Examination [MMSE] score < 10 pt.), delirium, terminal illness, severe neurologic, cardiovascular, metabolic or psychiatric disorders that compromised the ability to complete study procedures, and isolation for infection control. The sample size was determined according to a pragmatic criterion of maximizing the number of participants over the 8-month study period and allowing all patients admitted to the hospital who met the inclusion criteria to participate in the study. A sample size of ≥ 150 patients was intended to be achieved within the recruitment period.

### Acute geriatric care

The “early rehabilitative geriatric complex treatment” is an AGC model delivered in the acute hospital setting with ≥ 7 treatment days and 10 therapy sessions (OPS code 8-550.0), ≥ 14 treatment days and 20 therapy sessions (OPS code 8-550.1), or ≥ 21 treatment days and 30 therapy sessions (OPS code 8-550.2) provided by an interdisciplinary team under the direction of a geriatrician. Each therapy sessions lasts on average 30 min. Based on a comprehensive geriatric assessment and depending on the patients’ individual needs identified through it, a multidimensional treatment plan is developed that is based on patient-centered goals and covers at least two of the following therapeutic domains: physiotherapy, occupational therapy, speech/facio-oral tract therapy, and/or (neuro-)psychology. Once a week, an interdisciplinary team meeting is held with all health professionals involved in the treatment process, in which the results of the previous treatment outcomes and the further treatment steps and goals are discussed [26]. Supplementary Table 1 provides a systematic description of the early rehabilitative geriatric complex treatment using the Template for Intervention Description and Replication (TIDieR) checklist [38].

### Data collection

Data was collected as soon as possible after AGC prescription at hospital admission and after the end of AGC as close as possible at hospital discharge. All patient interviews and testing procedures were consistently administered by a physical therapist with 16 years of working experience who was well-trained in interviewing and testing geriatric patients and supported by a medical student to ensure patient safety.

#### Locomotor capacity

Locomotor capacity was assessed using the Short Physical Performance Battery (SPPB), consisting of hierarchical balance tests (side-by-side, semi-tandem and tandem stance), a 4-m gait speed (GS) test at usual pace, and a 5-chair stand test (5CST) [39]. Feasibility, construct validity, and predictive validity of the SPPB for functional decline, readmission, and/or mortality following hospitalization have been established in hospitalized older patients [40–42]. Meaningful changes have been estimated as ≥ 0.5 pt. (small) and ≥ 1 pt. (substantial) for SPPB and ≥ 0.05 m/s (small) and ≥ 1.0 m/s (substantial) for GS [43].

#### Physical activity

PA was measured using the uSense activity monitor, a small-scaled (42 × 10 × 68 mm) and lightweight (36 g) inertial measurement unit (accelerometer, gyroscope, and magnetometer) that was attached to the patients’ lower back (approximately at the height of the fifth lumbar vertebra) with waterproof adhesive foil. The uSense activity monitor and its non-commercial software for signal processing and feature extraction were developed in the FARSEEING project (Grant No. 288940, funded under the 7th Framework Programme of the European Union, FP7/2007-2013), and have been successfully validated in frail older adults [44] and geriatric patients [45]. The activity recognition software is able to detect the frequency and duration of four activity episodes (active, sedentary, walking, and lying), the number of steps, and mean daily PA level via metabolic equivalent of tasks (METs). An episode is labelled as “active” if METs are > 1.5 and as “sedentary” if METs are ≤ 1.5. Steps and walking are detected during active episodes based on acceleration data calculated by an adaptive algorithm, and lying during sedentary episodes if the trunk angle in medio-lateral or the anterior–posterior direction is below 30°. More details on the data processing of these PA parameters have been described elsewhere [45]. The uSense activity monitor was attached to the participants after study inclusion within the first 72 h of AGC initiation (median 3.2, IQR 0.7–22.9 h). They were instructed to wear the activity monitor continuously over 48 h. The initial 24-h activity recordings were used for analysis to determine participants’ PA behavior as early as possible after hospital admission and in the AGC treatment process. Patients with incomplete 24-h activity recordings within 4 days after AGC initiation (no or later measurement) were excluded. Towards the scheduled end of the AGC treatment period and hospital discharge, the uSense activity monitor was again attached to the participants for 48 h. For this follow-up assessment, the 24-h activity recordings closest to hospital discharge were used for analysis. For all PA assessments, only complete 24-h activity recordings on weekdays were used to describe PA.

#### Life-space mobility

The interview-based version of the Life-Space Assessment in Institutionalized Settings (LSA-IS) was used to assess patients’ LSM [46]. The LSA-IS documents the spatial extent of mobility, classified into five different zones within and around institutional settings (1 = own room, 2 = within the ward, 3 = within the facility, 4 = immediate outdoor area of the facility, 5 = beyond the area of the facility) and the frequency of mobility within each zone (1 × /day, 2–3 × /day, 4–5 × /day, 5 × /day) during the previous day, also taking into account the level of assistance needed for mobility (personal support, equipment, without any support). This information is combined to produce an LSA-IS total score (LSA-IS-T), ranging from 0 (bed-bound) to 120 pt. (complete independent mobility ≥ 5 × /day beyond the facility area). In addition, three sub-scores (each with a range 0–5 pt.) are determined for the maximum zone achieved with equipment or personal support if needed (LSA-IS-M), with equipment if needed but without personal support (LSA-IS-E), and independently without any equipment or personal support (LSA-IS-I). The LSA-IS has shown to be a valid, reliable, responsive, and feasible instrument for assessing LSM in acutely hospitalized older patients [46]. LSA-IS was administered at the beginning and the end of AGC for both days on which patients also wore the uSense activity monitor to capture the same time period PA was recorded. Again, the recording days closest to the hospital admission and discharge were used for the analysis.

#### Other characteristics

Age, gender, primary (reason for admission) and secondary diagnoses (comorbidities), medications, and living situation before admission (community-dwelling, assisted living facility, or nursing home), functional status in ADLs (Barthel Index, BI), and length of hospital stay (LOS) were documented from patient charts. Cognitive status (MMSE), depressive symptoms (Geriatrics Depression Scale, 15-item version, GDS-15), FoF (Short Falls Efficacy Scale-International, FES-I) [47], falls in the previous years, primary mode of locomotion (independent walking, walking with an assistive mobility device, wheelchair dependent), nutritional status (Body Mass Index, BMI) and frailty were assessed by standardized patient interviews or testing procedures. Frailty was determined according to the Fried frailty phenotype and having 3 or more of: (1) unintentional weight loss (> 4.5 kg in the past year), (2) self-reported exhaustion (2-items from the Center for Epidemiological Survey-Depression Scale), low PA (short version of the Minnesota Leisure Time Physical Activity Questionnaire: female < 270 kcal/week, male < 383 kcal/week), slowness (gender- and height-adjusted slow GS), and weakness (gender- and BMI-adjusted low maximum handgrip strength as measured with a JAMAR hydraulic hand dynamometer) [48].

### Statistical analysis

Descriptive data was given as frequencies and percentages, medians and interquartile ranges (IQR), or means and standard deviations (SD). Changes in mobility outcomes and other variables over the AGC treatment period were analyzed using Wilcoxon signed-rank tests. Effect sizes were calculated as *r* = (z/√*n*) and interpreted as small (*r* < 0.3), moderate (0.3 ≤ *r* < 0.5), or large (*r* ≥ 0.5). Chi-square tests and Mann–Whitney *U*-tests were used to compare differences between dropouts after baseline assessment and patients who completed the study. To identify potential factors predictive of mobility outcomes (SPPB, step count, LSA-IS-T) at discharge, separate univariate linear regression models adjusted for the baseline value of each mobility outcome were performed. Step count was naturally log-transformed before regression analyses due to non-normally distributed residuals. The candidate variables analyzed for step count and LSA-IS-T at discharge included age, sex, and nutritional status (underweight: BMI < 23 kg/m^2^, normal weight: BMI = 23–30 kg/m^2^, overweight: BMI > 30 kg/m^2^) [49], medications, comorbidities, cognitive impairment (MMSE < 24 pt.), depressive symptoms (GDS-15 > 5 pt.) [50], FoF (low: FES-I = 7–8 pt., moderate: FES-I = 9–14 pt., high: FES-I ≥ 14 pt.) [51], frailty, primary locomotion mode, locomotor capacity (SPPB), and functional status (BI) at admission. For the SPPB at discharge, PA (step count, activity duration [active + walking duration], mean daily PA level, mean walking bout duration), and LSM (LSA-IS-T) were also analyzed as candidate variables. Candidate variables were based on previous findings on predictors of functional and mobility outcomes from the literature (see introduction). All variables with a *p*-value of < 0.10 in the univariate analyses were entered into a multivariable linear regression model to identify independent predictors. Beta weights and *p*-values are reported for the results of the regression models. As the primary diagnosis for admission was not considered in the original regression analyses but could be a confounding factor, an additional sensitivity analysis was conducted in which the multivariable regression models were adjusted for the diagnosis. *P*-values of < 0.05 were considered statistically significant. All statistical analyses were performed using IBM Statistics for Windows, Version 27.0 (IBM Corp., Armonk, NY, USA).

## Results

### Baseline patient characteristics

**Fig. 1:**
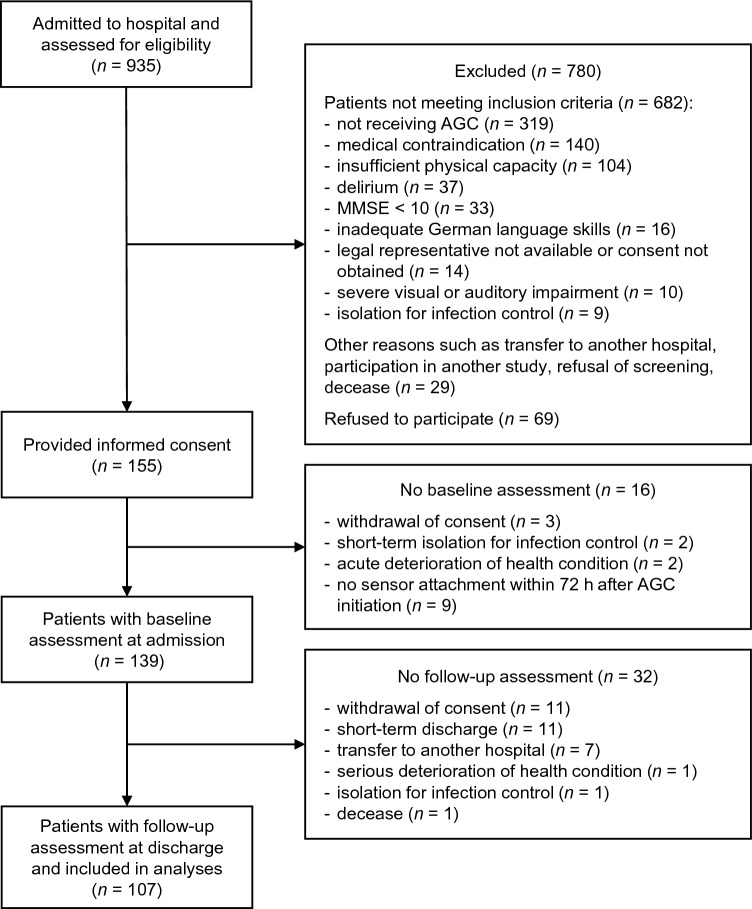
Flowchart of the recruitment, screening, baseline and follow-up assessment, and data analysis

The flow of the patient recruitment to data analysis is illustrated in Fig. 1. Out of 935 patients admitted to the hospital and screened for eligibility during the recruitment period, 155 gave written informed consent to participate in the study. Baseline assessment was performed with 139 patients, of whom 107 underwent the follow-up assessment at the end of AGC and were finally included in the data analyses. Main reasons for dropout were consent withdrawal, short-term discharge, and transfer to another hospital. Dropouts after baseline assessments (*n* = 32) did not differ significantly from study completers in any mobility outcomes (*p* = 0.103–0.983) or other patient characteristics assessed at baseline (*p* = 0.052–0.898), except for fall history (dropouts: 63% fallers vs. completers: 81% fallers; *p* = 0.041). Mean LOS of patients included in the analyses was 20.2 ± 5.8 days.

The sample included 107 multi-morbid (diagnoses = 9.7 ± 5.4), and older patients (age = 83.2 ± 6.4 years) (Table 1). Over 60% were identified as being frail, more than 50% had cognitive impairment, and about 40% showed depressive symptoms. More than 80% of patients reported ≥ 1 fall in the previous year and about two-thirds (64.5%) showed moderate to high FoF. Functional status was severely impaired, with a median (IQR) BI of 55 (45–70) pt.

**Table 1:** Baseline patient characteristics

Variable	*n* = 107^a^
Age, years	83.2 ± 6.4
Female, *n*	68 (63.6)
Living situation before admission, *n*	
Community-dwelling	94 (87.9)
Assisted living	11 (10.3)
Nursing home	2 (1.9)
BMI, kg/m^2^	26.0 ± 5.0
Underweight (< 23 kg/m^2^)	31 (29.0)
Normal (23–30 kg/m^2^)	57 (53.3)
Overweight (> 30 kg/m^2^)	19 (17.8)
Primary diagnosis for admission, *n*	
Musculoskeletal	32 (29.9)
Neurological	18 (16.8)
Infectious	12 (10.3)
Cardiovascular	9 (8.4)
Gastrointestinal	9 (8.4)
Neuromusculoskeletal	6 (5.6)
General health deterioration	6 (5.6)
Others	15 (14.0)
Comorbidities, *n*	9.7 ± 5.2
Medications, *n*	10.0 ± 3.9
LOS, days	20.2 ± 5.8
≥ 1 fall in the previous year, *n*	88 (81.5)
MMSE, pt	22.3 ± 4.8
Cognitive impairment, *n* (MMSE < 24 pt.)	59 (55.1)
GDS-15, pt	5.1 ± 3.5
Depressive symptoms, *n* (GDS > 5 pt.)	42 (39.3)
Short FES-I, pt. (*n* = 104)	11 [8–17]
Low FoF (7–8 pt.)	35 (32.7)
Moderate FoF (9–13 pt.)	28 (26.2)
High FoF (≥ 14 pt.)	41 (38.3)
Primary locomotion mode, *n*	
Independent walking	17 (15.9)
Walking with AMD	77 (72.0)
Wheelchair dependent	13 (12.1)
Frailty, *n* (*n* = 99)	66 (61.7)
Barthel Index, pt	55 [45–70]
SPPB, pt. (*n* = 104)	4.0 ± 2.2
Gait speed, m/s (*n* = 104)	0.42 ± 0.23
5CST, s (*n* = 69)	21.1 [18.1–37.1]
Handgrip strength, kg (*n* = 103)	16.6 ± 7.7
Physical activity (*n* = 102)	
Active, min	30.2 [17.5–48.7]
Walking, min	6.6 [1.2–19.1]
Sedentary, min	601.0 [412.6–692.4]
Lying, min	797.1 [685.4–971.0]
Step count, *n*	516 [89–1806]
Mean walking bout duration, s	7.9 [4.9–11.4]
LSA-IS total score, pt. (*n* = 106)	10.8 [6.8–15.0]

*BMI* Body Mass Index, *LOS* length of hospital stay, *MMSE* Mini-Mental State Examination, *GDS-15* Geriatric Depression Scale-15 item version, *FES-I* Falls Efficacy Scale-International, *FoF* Fear of Falling, *AMD* assistive mobility device, *SPPB* Short Physical Performance Battery, *5CST* 5-chair stand test, *LSA-IS* Life-Space Assessment in Institutionalized Settings

^*a*^*n* = 107, unless otherwise indicated. Data are given as *n* (%), median [IQR], or mean ± SD

Patients’ baseline mobility was very limited. Locomotor capacity was low: SPPB score averaged 4.0 ± 2.2 pt., usual GS 0.42 ± 0.23 m/s, and maximum handgrip strength 16.6 ± 7.7 kg. Median 5CST duration was 21.1 [18.1–37.1] s, with about one-third (*n* = 35, 33.7%) of patients unable to complete an initial single chair stand. PA levels were also very low, with patients spending a median of 1403.2 (IQR 1364.7–1418.0) min inactive (sedentary/lying) and 36.6 (IQR 22.0–75.3) min (active/walking) during the day. A median of only 6.6 (IQR 1.2–19.1) min per day was spent walking. Median daily step count was 516 (IQR 89–1806) steps and the median daily walking bout duration was 7.9 (IQR 4.9–11.4) s. The median LSA-IS-T score of 10.8 (IQR 6.8–15.0) pt. indicated a very restricted LSM. About one-third (32.1%, *n* = 34) of patients did not leave their own room without personal support (LSA-IS-E ≥ 2 pt.). Independent mobility in their own room without any assistance (LSA-IS-I ≥ 1 pt.) was observed in only 14.2% (*n* = 15) of patients. Only 22.6% (*n* = 24) moved outside their own ward, even with the use of equipment or personal support (LSA-IS-M ≥ 3 pt.). Outdoor mobility was observed in only 6.6% (*n* = 7, LSA-IS-M ≥ 4 pt.).

### Treatment effects

The SPPB total score (% median change =  + 25.0%), usual GS (+ 16.3%), and 5CST duration (− 12.2%) significantly improved over AGC (*p* < 0.001) (Table 2). Median improvements of 1 pt. in the SPPB and 0.09 m/s in GS were clinically meaningful. Active (+ 36.1%) and walking duration (+ 104.9%), mean daily PA level (+ 2.1%), step count (+ 115.3%), and mean walking bout duration (+ 20.3%) were also significantly increased at discharge (*p* < 0.001–0.009). More than half (58.8%, *n* = 55) of patients showed an increased step count of ≥ 100 steps. All LSA-IS scores also significantly increased (*p* < 0.001–0.026). The proportion of patients not leaving their own room without personal support halved to one-sixth (16.6%, *n* = 17, LSA-IS-E ≤ 1 pt.). Inside their own room, about one-third (32.4%, *n* = 33) moved independently without any assistance (LSA-IS-I ≥ 1 pt.) at the end of AGC. LSM outside their own ward increased to 48.0% of patients (*n* = 49, LSA-IS-M ≥ 3 pt.) and outdoor mobility to 29.4% (*n* = 30, LSA-IS-M ≥ 4 pt.). Highest effect sizes within the distinct mobility constructs were observed for GS (*r* = 0.405), mean daily PA level (*r* = 0.536), and LSA-IS-T (*r* = 0.652). Effect sizes for significant improvements in locomotor capacity were moderate (*r* = 0.349–0.405), while those in PA (*r* = 0.431–0.536) and LSM (*r* = 0.447–0.652) were also partly even large.

**Table 2:** Changes in locomotor capacity, physical activity, and life-space mobility over acute geriatric care

Variable	Admission	Discharge	Δ	*p*	Effect
*Locomotor capacity*					
SPPB, pt. (*n* = 98)	4.0 [2.8–5.0]	5.0 [3.0–6.3]	1.0 [0.0–2.0]	< 0.001	0.354
Balance test, pt. (*n* = 98)	2 [1–3]	2 [1–3]	0 [0–1]	0.148	0.143
Gait speed, m/s (*n* = 98)	0.43 [0.25–0.58]	0.50 [0.29–0.70]	0.09 [–0.04, 0.18]	< 0.001	0.405
5CST, s (*n* = 56)	20.5 [17.8–32.2]	18.0 [14.7–23.9]	–1.9 [–5.7, –0.3]	< 0.001	0.349
Handgrip strength, kg (*n* = 96)	16.0 [12.0–21.5]	16.0 [11.0–21.8]	0.0 [–2.0, 3.0]	0.207	0.125
*Physical activity* (*n* = 92)					
Total duration, min					
Active	30.2 [18.1–48.9]	41.1 [25.6–62.6]	7.7 [–2.4, 26.1]	< 0.001	0.431
Sedentary	597.8 [411.8–691.7]	596.4 [456.9–712.4]	29.4 [–113.9, 117.2]	0.464	0.076
Walking	6.1 [1.2–21.7]	12.5 [2.7–39.6]	3.0 [–0.5, 13.4]	< 0.001	0.473
Lying	803.0 [689.2–968.6]	770.5 [655.0–915.4]	–41.6 [–146.9, 77.3]	0.110	0.167
Mean daily PA level, METs	1.45 [1.40–1.50]	1.48 [1.43–1.53]	0.02 [–0.01, 0.06]	< 0.001	0.536
Step count, *n*	516 [89–1806]	1111 [228–3291]	224 [–64, 1289]	< 0.001	0.452
Mean walking bout duration, s	7.9 [5.0–11.3]	9.5 [6.9–13.4]	1.2 [–1.4, 4.2]	0.009	0.273
*Life-space mobility* (*n* = 102)					
LSA-IS, pt.					
Total score	10.5 [6.0–15.0]	16.3 [12.0–24.1]	6.0 [1.4–10.6]	< 0.001	0.652
Maximum sub-score	2 [2–2]	2 [2–4]	0 [0–1]	< 0.001	0.520
Equipment-assisted sub-score	2 [1–2]	2 [2–3]	0 [0–1]	< 0.001	0.447
Independent sub-score	0 [0–0.3]	0 [0–1]	0 [0–0]	0.026	0.221

Data are presented as median [IQR]. *p*-values are given for Wilcoxon signed-rank tests. Effect sizes were calculated as *r* = (z/√*n*)

*SPPB* Short Physical Performance Battery, *5CST* 5-chair stand test, *PA* physical activity, *MET* metabolic equivalent of task, *LSA-IS* Life-Space Assessment in Institutionalized Settings

Further significant improvements in other patient characteristics were observed for the BI (+ 27.3%), the MMSE (+ 4.3%), and the FES-I (− 9.1%) (*p* < 0.001–0.019; Supplementary Table 2).

### Predictors of mobility outcomes

Initial univariable analyses showed that female gender (*ß* = –0.825, *p* = 0.029), lower nutritional status (underweight vs. normal weight: *ß* = –0.846, *p* = 0.041), and mean daily PA level (*ß* = –4.144, *p* = 0.088) were inversely associated with the SPPB score at discharge at a significance level of *p* < 0.10, independent of the SPPB at admission (simple regression: *ß* = 0.839, *p* < 0.001) (Table 3). When these variables were entered into the multivariable linear regression model, non-frailty (*ß* = –1.103, *p* = 0.005), a higher mean daily PA level (*ß* = 0.635, *p* = 0.027), and a higher SPPB score at admission (*ß* = 0.689, *p* = 0.001) were identified as independent positive predictors of the SPPB score at discharge. This model explained 60.4% of the variance in the SPPB score at discharge.

**Table 3:** Univariable and multivariable linear regression analyses for locomotor capacity at hospital discharge

Variable	SPPB at discharge			
	Univariable analysis^a^		Multivariable analysis	
	*ß*	*p*	*ß*	*p*
Age	− 0.027	0.340		
Gender^b^	− 0.825	0.029	− 0.687	0.066
BMI				
Normal (ref.)	–	–		
Underweight	− 0.846	0.041	− 0.783	0.057
Overweight	− 0.499	0.316	− 0.455	0.338
Comorbidities	− 0.037	0.302		
Medications	− 0.013	0.789		
Cognitive impairment	− 0.205	0.576		
Depressive symptoms	− 0.498	0.233		
FoF				
Low (ref.)	–	–		
Moderate	− 0.018	0.969		
High	− 0.388	0.393		
Primary locomotion mode				
Independent walking (ref.)				
Walking with AMD	− 0.364	0.520		
Wheelchair dependent	− 1.155	0.207		
Frailty	− 0.931	0.020	− 1.103	0.005
Barthel Index	0.011	0.329		
Physical activity				
Step count^c^	0.076	0.520		
Activity duration	0.008	0.371		
Mean daily PA level^d^	0.046	0.088	0.635	0.027
Mean walking bout duration	1.224	0.617		
LSA-IS-T	− 0.012	0.663		
SPPB at admission	0.839	< 0.001	0.689	< 0.001
			Adjusted *R*^*2*^ = 0.604	

*BMI* Body Mass Index, *FoF* fear of falling, *AMD* assistive mobility device, *PA* physical activity, *LSA-IS-T* Life-Space Assessment in Institutionalized Settings, total score, *SPPB* Short Physical Performance Battery

^a^Except for the regression coefficient for baseline values (simple regression), coefficients for the different variables were adjusted for baseline values (step count or LSA-IS-T at admission)

^b^Female = 0, male = 1

^c^Natural log-transformed

^d^*ß* given for an increase of 0.01 METs

Univariable analyses for the step count at discharge revealed significant associations with the primary locomotion mode (wheelchair dependent vs. independent walking: *ß* =  − 1.840, *p* = 0.004), frailty (*ß* =  − 0.898, *p* = 0.007), BI (*ß* = 0.032, *p* < 0.001), and SPPB (*ß* = 0.220, *p* = 0.005), independent of step count at admission (simple regression: *ß* = 0.551, *p* < 0.001) (Table 4). In the multivariable linear regression model, non-frailty (*ß* =  − 0.676, *p* = 0.033), a higher BI (*ß* = 0.032, *p* < 0.001), and a higher step count at admission (*ß* = 0.307, *p* = 0.002) were independently associated with a higher step count at discharge. The proportions of the variance in step count at discharge explained by this model was 47.7%.

**Table 4:** Univariable and multivariable linear regression analyses for physical activity and life-space mobility at hospital discharge

Variable	Step count^a,b^ at discharge				LSA-IS-T at discharge			
	Univariable analysis^a^		Multivariable analysis		Univariable analysis^b^		Multivariable analysis	
	*ß*	*p*	*ß*	*p*	*ß*	*p*	*ß*	*p*
Age	− 0.018	0.454			− 0.184	0.233		
Gender^c^	0.122	0.696			− 2.698	0.167		
BMI								
Normal (ref.)								
Underweight	− 0.093	0.800			− 0.209	0.924		
Overweight	− 0.246	0.539			− 1.600	0.535		
Comorbidities	− 0.031	0.292			− 0.026	0.886		
Medications	− 0.012	0.757			− 0.001	0.998		
Cognitive impairment	− 0.126	0.680			− 4.144	0.028	− 2.932	0.124
Depressive symptoms	0.220	0.485			− 1.896	0.324		
FoF								
Low (ref.)								
Moderate	− 0.158	0.678			0.430	0.864		
High	− 0.549	0.134			0.415	0.858		
Primary locomotion mode								
Independent walking (ref.)								
Walking with AMD	− 0.300	0.469	− 0.269	0.580	1.521	0.565		
Wheelchair dependent	− 1.840	0.004	− 1.548	0.051	− 4.588	0.229		
Frailty	− 0.898	0.007	− 0.676	0.033	− 7.129	0.001	− 6.346	0.003
Barthel Index	0.032	< 0.001	0.023	0.017	0.082	0.126		
SPPB	0.220	0.005	0.032	0.725	0.733	0.142		
Baseline values								
Step count^a,b^	0.551	< 0.001	0.307	0.002				
LSA-IS-T	–	–			0.662	< 0.001	0.603	< 0.001
			Adjusted *R*^*2*^ = 0.477				Adj﻿us﻿ted *R*^*2*^ = 0.323	

*BMI* Body Mass Index, *FoF* fear of falling, *AMD* assistive mobility device, *LSA-IS-T* Life-Space Assessment in Institutionalized Settings, total score, *SPPB* Short Physical Performance Battery

^a^Except for the regression coefficient for baseline values (simple regression), coefficients for the different variables are adjusted for baseline values (step count or LSA-IS-T at admission)

^b^Natural log-transformed. ^c^Female = 0, male = 1

In the univariable analyses for the LSA-IS-T at discharge, significant associations were found with cognitive impairment *(ß* =  − 4.144, *p* = 0.028) and frailty (*ß* =  − 7.129, *p* = 0.001), independent of the LSA-IS-T at admission (simple regression: *ß* = 0.662, *p* < 0.001) (Table 4). The multivariable linear regression model revealed that non-frailty (*ß* =  − 6.346, *p* = 0.003) and a higher LSA-IS-T at admission (*ß* = 0.603, *p* < 0.001) were independently associated with a higher LSA-IS-T score at discharge. This model explained 32.3% of the variance in the LSA-IS-T at discharge.

**Fig. 2:**
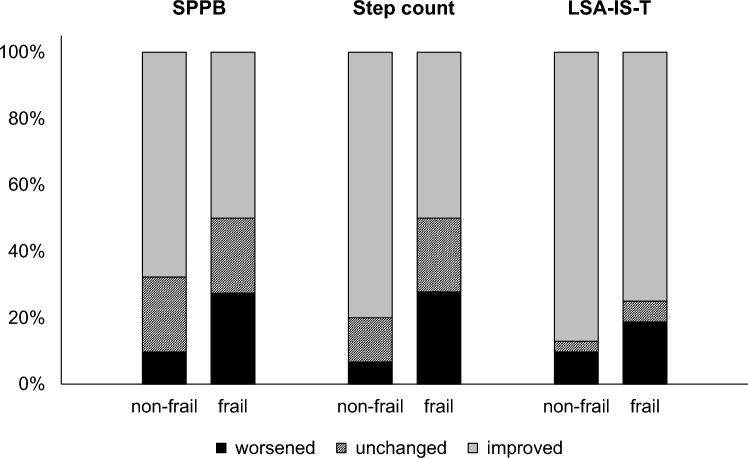
Distributions of improved (grey), unchanged (hatched), and worsened (black) patients in the SPPB (≥ + 1 vs. 0 vs. ≤ –1 pt.), step count (≥ + 100 vs. + 99 to -99 vs. ≤ –100 steps), and LSA-IS-T (≥ + 1 vs. 0 vs. ≤ –1 pt.), stratified by frailty status

The proportion of participants with a meaningful change of 1 pt. in the SPPB, a 100-step change in step count, and a 1-point change in LSA-IS-T is shown in relation to the frailty status in Fig. 2. In all three mobility outcomes, improvements were more frequent in non-frail patients and deterioration were more frequent in frail patients.

Sensitivity analysis with the multivariable regression models adjusted for primary admission diagnosis confirmed results, showing that (1) frailty and mean daily PA level were predictive of SPPB at discharge, (2) frailty, BI, and primary locomotion mode (independent walking vs. wheelchair dependent) were predictive of step count at discharge, and (3) frailty were predictive of LSA-IS-T at discharge, independent of baseline SPPB, step count, and LSA-IS-T, respectively, and diagnosis (Supplementary Table 3–5).

## Discussion

The results of this study demonstrated that hospitalized older patients benefited from AGC in distinct mobility outcomes. AGC resulted in clinically meaningful improvements in locomotor capacity (SPPB, GS) and significantly increased physically active behavior (active and walking duration, daily PA level, step count, walking bout duration) and LSM (LSA-IS-T) at hospital discharge. To our knowledge, this is the first study to provide insight into longitudinal changes in distinct mobility outcomes among older patients undergoing AGC in hospital settings. Frailty was the only independent factor consistently found to be negatively predictive of outcomes in all mobility outcomes (locomotor capacity, PA, LSM).

Patients showed very low locomotor capacity at the beginning of the AGC, with a median SPPB total score of 4 pt. and a median usual GS of 0.43 m/s. This is in line with findings from other studies that assessed the locomotor capacity of older patients early after admission to AGC hospital wards [52, 53]. Such low locomotor capacity has been associated with several adverse health outcomes in old age, such as disability, institutionalization, falls, and/or mortality [39, 54]. AGC effectively improved patients’ locomotor capacity, as indicated by clinically meaningful improvements on a population level in the SPPB (median change =  + 1 pt.), as a composite measure for basic daily mobility tasks, and in walking capacity (median change in GS =  + 0.09 m/s), as the most fundamental form of human locomotion. This finding suggests the physical resilience of multi-morbid older patients with acute medical conditions and the potential of AGC to enhance the locomotor capacity of this vulnerable patient population, which is consistent with previous studies [27, 28, 35]. A 1-point improvement in the SPPB during (post-)acute geriatric care has been associated with about 20% lower risk of mortality and institutionalization within three months after hospital discharge in older patients [55], and each 1-point higher SPPB at hospital discharge with a 13% [40] and 14% [41] lower risk of hospital readmission and/or mortality, respectively, and an 18% lower risk of functional decline after one year [41]. Similar positive findings have been reported for 0.1-m/s improvements in GS among hospitalized older patients, with reduced risks for readmission of 65%, institutionalization of 73%, and mortality of 80% one year after discharge [55]. Despite the meaningful improvements during AGC, however, it needs to be acknowledged that patients’ locomotor capacity at discharge was still very low (median [IQR] SPPB = 5.0 [3.0–6.3] pt., GS = 0.50 [0.29–0.70] m/s), which remains a relevant risk factor for subsequent adverse health outcomes after hospital stay [40, 41].

Also very low levels of PA were observed at the beginning of AGC, with over 95% of the day being inactive, less than 7 min in median of walking, and only 516 steps taken during the day. These findings are consistent with those from previous studies reporting physically active behavior < 6% per day [5], median daily walking duration of 4 min [8], and median/mean daily step counts of 222 to 541 steps for the early phase after hospital admission in older patients undergoing AGC [4, 5, 8]. Patients significantly increased their PA over hospital stay. Walking duration (+ 105%) and number of steps were found to be more than doubled (+ 115%) at AGC treatment. Such substantial increases in walking behavior over the AGC treatment period has also been reported in previous studies in comparable patient populations [5, 8]. About 60% of our patients increased their step count by ≥ 100 steps. A previous study found that in patients undergoing AGC, each 100-step increment in the last 24 h of hospitalization after AGC was associated with a 3% lower 2-year mortality risk [5]. There was also a significant increase in the mean walking bout duration as a potentially more capacity-related PA parameter (+ 20%), though it still appears to be relatively short (median 9.5 s). To our knowledge, this parameter has not yet been investigated in acutely hospitalized older adults.

Patients initially showed a very restricted LSM, as measured with the recently developed LSA-IS [46]. Comparison with findings of previous studies is hampered due to other assessment tools used to describe LSM, focusing mainly on the spatial extent rather than frequency and/or need of assistance to move within the hospital setting, and/or due to not clearly defined timing of data collection in the treatment process [11–13]. Previous studies have reported that only 19–27% of acutely hospitalized older patients moved within the hallways of their wards [11, 12], which is considerably lower than the proportion in our sample (68%). This might be due to the fact that even though these studies were also conducted in acute care hospitals, the patients did not receive AGC with early mobilization and activation to promote patients’ (life-space) mobility. The LSA-IS total (median change =  + 6 pt. [+ 55%]) and all sub-scores (maximum, equipment-assisted, independent) were significantly improved after AGC, indicating that the spatial extent and/or frequency of LSM increased and/or need of assistance for mobility decreased. An improvement of 6 pt. in the LSA-IS-T corresponds, for example, to a change in patients’ LSM from moving once daily within the ward with personal support to moving two to three times daily within the ward with equipment, from moving only indoors to moving once daily outdoors with equipment, or from moving once daily within the own ward to moving once daily within the hospital daily without equipment or personal support. To our knowledge, such improvements in the LSM during AGC has not yet been reported. Our findings suggest that patients were enabled to overcome some personal barriers to LSM and to move more independently and/or frequently in a larger life space, which is a prerequisite for subsequent activity and participation in daily life after hospital discharge.

Significant positive effects of AGC were observed for SPPB and GS, but interestingly, those for PA and LSM were higher. This improvement in real-world mobility may be more crucial from an overall health perspective to regain activity and participation in daily life after hospital discharge.

Frailty was the only factor consistently identified as an independent negative predictor of all distinct mobility outcomes (SPPB, step count, LSA-IS-T). Our results add to the previous evidence that frail compared to non-frail older patients not only might show poorer gains in locomotor capacity from AGC [35], but also in PA and LSM, which both more closely refer to a person’s mobility behavior in the real-world environment after hospital discharge. The additional independent association between baseline BI and increased PA at discharge underscores the less benefit from AGC in more vulnerable patients with difficulty in ADL functioning, which often co-occur with frailty. These findings may have potential implications for optimizing AGC for more vulnerable persons by incorporating more frailty-specific intervention components to ensure AGC to benefit frail as much as non-frail patients with respect to positive mobility outcomes.

Previous studies have shown that higher accelerometer-measured PA levels over hospital stay, as quantified by averaged steps and/or activity duration per day, were associated with improvements in ADL functioning among older patients admitted to internal medicine wards [16, 17]. Contrary to these studies, we did not analyze an average PA level over several treatment days or the total hospital stay as a potential predictor but focused on PA measured as early as possible after AGC initiation to examine the potential impact of early mobilization and activation, as one main goal of AGC, on locomotor capacity. Our results showed no significant association of SPPB changes with a number of steps or activity duration at hospital admission but with overall 24-h PA level. A higher PA level was identified to be independently predictive of SPPB improvements after AGC. A similar positive association between accelerometer-measured PA levels at admission with improvements in locomotor capacity (SPPB) has been observed in older adults undergoing post-acute hospital rehabilitation [34]. Considering that physical inactivity can rapidly lead to extraordinary loss of muscle strength in older adults [14] and the SPPB is closely related to lower extremity muscle strength, our finding underscores the significant benefit of promoting PA in the early phase of AGC for patients' locomotor capacity and the relevance of early mobilization as part of AGC.

This study has some limitations. First, patients with severe cognitive (MMSE < 10 pt., delirium) and gait impairments (inability to walk ≥ 4 m with walking aid) were excluded, and thus the results are not generalizable to such more affected populations. Second, the study had a single-center observational design, and mobility outcomes and associated factors were analyzed in patients receiving “early rehabilitative geriatric complex treatment” as an AGC model routinely provided in Germany. Naturally, our findings may be influenced by the specific structure of the German healthcare system, which may limit their generalizability to other countries. Third, baseline data were collected as early as possible after AGC initiation, which did not always correspond to the first day of patients’ hospital stay, due to decision-making processes about prescription appropriateness for AGC that required some time or to organizational reasons (e.g., admission on Fridays, AGC prescription on Mondays). Fourth, due to limited technical (e.g., sensor availability, battery life) and personnel resources, and to avoid compliance issues, PA and LSM were assessed only twice at the beginning and end of the AGC. Future technical developments of sensors with longer battery life, smaller size and less costs may allow continuous PA monitoring during the entire hospital stay with high patient acceptance. Fifth, PA and LSM might have been influenced by the daily routines in the AGC wards, which are very structured and where mobility behavior is partly predetermined by the individual therapy plan. We were not able to distinguish self-initiated from therapy-induced PA and LSM. Future studies are needed to examine the impact of such external factors on mobility behavior. Finally, although independent associations were found between the predictors used in the regression models and mobility outcomes, no causal relationships can be interpreted.

In conclusion, the results of the PAGER study show that acutely hospitalized older patients benefit from AGC in distinct mobility constructs (locomotor capacity, PA, LSM). Frailty was consistently identified as an independent negative predictor of all these mobility constructs after AGC. This finding suggests that routine frailty assessment in AGC is important to identify patients at risk for decreased treatment gains in mobility. It supports future studies to determine how AGC can be adapted to better match the specific needs of hospitalized older patients with more advanced frailty and optimize their mobility outcomes. Early PA promotion in AGC seems to be beneficial in enhancing AGC patients’ locomotor capacity at hospital discharge.

### Supplementary Information

Below is the link to the electronic supplementary material.

Supplementary file1 (DOCX 33 KB)

## Data Availability

The datasets used and analyzed during the current study are available from the corresponding author on reasonable request.
