# Supplementary material for: Mobility outcomes and associated factors of acute geriatric care in hospitalized older patients: results from the PAGER study
Source: Eur Geriatr Med. 2023 Oct 1;15(1):139–52. doi: 10.1007/s41999-023-00869-9 (PMC10876756; doi:10.1007/s41999-023-00869-9)
Supplement: Supplementary file 1 — Supplementary file1 (DOCX 33 KB) [file 41999_2023_869_MOESM1_ESM.docx]

*Title*

**Mobility outcomes and associated factors of acute geriatric care in hospitalized older patients: Results from the PAGER study**

*Journal*

**European Geriatric Medicine**

Christian Werner,^1,*^ Laura Bauknecht,^2^ Patrick Heldmann,^3,4^ Saskia Hummel,^2^ Michaela Günther-Lange,^1^ Jürgen M, Bauer,^1,3^ Klaus Hauer^1,5^

^1^ Geriatric Center, Heidelberg University Hospital, Agaplesion Bethanien Hospital Heidelberg, Rohrbacher Str. 149, 69216 Heidelberg, Germany.

^2^ Medical Faculty Heidelberg, Heidelberg University, Im Neuenheimer Feld 672, 69120 Heidelberg, Germany.

^3^ Network Aging Research (NAR), Heidelberg University, Bergheimer Str. 20, 69115 Heidelberg, Germany.

^4^ Division of Physiotherapy, Department of Applied Health Sciences, Hochschule für Gesundheit (University of Applied Sciences), Gesundheitscampus 6-8, 44801 Bochum, Germany.

^5^ Department of Clinical Gerontology, Robert-Bosch-Hospital, Auerbachstraße 110, 70376 Stuttgart, Germany.

***Corresponding author**

Dr. Christian Werner

Tel: +49 6221 319 1759

E-mail: [christian.werner@agaplesion.de](mailto:christian.werner@agaplesion.de)

**Supplementary Table 1.** Description of the “early rehabilitative geriatric complex treatment” using the TIDieR checklist.

| **Item** | **Description** |
| --- | --- |
| NAME | Early rehabilitative geriatric complex treatment |
| WHY | Low physical activity (e.g., prolonged bed rest) is highly prevalent in older adults acutely admitted to hospital, and increases the risk of various negative outcomes, such as hospital-acquired functional decline, longer hospital stay, readmission to hospital, institutionalization, and mortality. Acute geriatric care models such as the “early rehabilitative geriatric complex treatment” aim to maintain and enhance patients’ independence in activities of daily living and mobility, and to prevent hospital-acquired functional decline through early mobilization and rehabilitation. The benefits of acute geriatric care models on functioning in activities of daily living have already been well documented; however, those on distinct mobility outcomes (locomotor capacity, physical activity, and life-space mobility) are not yet known. |
| WHAT | The main components of the “early rehabilitative geriatric complex treatment” included:   - Standardized geriatric assessment at the beginning and at the end of the treatment process in at least four domains (mobility, functional independence, mobility, cognition, emotions). - Social assessment on previous status in at least five domains (social environment, living environment, home/out-of-home activities, care/assistance needs, legal dispositions). - weekly interdisciplinary team meeting with the treatment-leading geriatrician and at least one representative of the nursing staff, and the therapeutic domains of physiotherapy, occupational therapy, speech therapy/facio-oral tract therapy, and(neuro-)psychology. Here, the result of the previous treatment outcomes and the further treatment steps and goals were discussed. - Activating-therapeutic care by specially trained, geriatric nurses. - Multidimensional treatment plan of at least two of the following four therapeutic domains: physiotherapy, occupational therapy, speech therapy/facio-oral tract therapy, and/or (neuro-)psychology. |
| WHO PROVIDED | Interdisciplinary geriatric team led by a geriatrician and consisting of physiotherapists, occupational therapists, psychologists, geriatric nurses, and social workers. |
| HOW | At least 9 out of 10 (OPS code 8-550.0), ≥18 out of 20 (OPS code 8-550.1), or ≥27 out of 30 (OPS code 8-550.2), therapy sessions provided in 1:1 therapist-patient ratio. |
| WHERE | Three acute geriatric wards of the Agaplesion Bethanien Hospital Heidelberg, Geriatric Centre at Heidelberg University Hospital (Heidelberg, Baden-Württemberg, Germany). |
| WHEN AND HOW MUCH | Therapy sessions were delivered on weekdays, and started as early as possible after hospital admission. Treatment lasted ≥7 days with 10 therapy sessions (OPS code 8-550.0), ≥14 days with 20 therapy sessions (OPS code 8-550.1), or ≥21 days with 30 therapy sessions (OPS code 8-550.2) until hospital discharge. Therapy sessions lasted on average 30 minutes and covered at least two of the following therapeutic areas, depending on the individual needs and goals of the patients: physiotherapy, occupational therapy, speech therapy/facio-oral tract therapy, and/or (neuro-)psychology. |
| TAILORING | Early rehabilitative geriatric complex treatment as the official acute geriatric care model according to the OPS in the German inpatient sector was standardized but its implementation was tailored to the individual needs and goals of the patients. The comprehensive geriatric assessment was used to identify functional deficits and to develop and implement a specifically tailored, coordinated and integrated treatment plan. |
| MODIFICATIONS | N/A |
| HOW WELL | Length of hospital stay was a median of 20 days [IQR 16-21], and therapy minutes received up to the assessment at hospital discharge was a median of 600 minutes [IQR 540-690], as documented in the electronic hospital records. Ninety-one (85.0%) patients received the "early rehabilitative geriatric complex treatment " according to OPS code 8-550.1, 13 (12.1%) patients according to OPS code 8-550.0, and 3 (2.8%) patients according to OPS code 8-550.2. |
| OPS, Operation and Procedure Classification System; IQR, interquartile range. | |

**Supplementary Table 2.** Changes in functional, cognitive and psychological outcomes over acute geriatric care.

| **Variable** | **Admission** | **Discharge** | **Δ** | ***p*** | **Effect** |
| --- | --- | --- | --- | --- | --- |
| Barthel Index, pt. (*n* = 107) | 55 [45-70] | 70 [50-85] | 5 [0-20] | <0.001 | 0.464 |
| MMSE, pt. (*n* = 103) | 23 [19-26] | 24 [21-27] | 0.5 [−1.0, 3.0] | 0.022 | 0.125 |
| GDS-15, pt. (*n* = 99) | 5 [2-7] | 4 [2-7] | 0 [−2, 1] | 0.202 | 0.097 |
| FES-I, pt. (*n* = 101) | 11 [8-17] | 10 [8-13] | −1 [−4, 2] | 0.008 | 0.283 |
| Data are presented as median and interquartile ranges. *p*-values are given for Wilcoxon signed-rank tests. Effect sizes were calculated as *r =* (z/√*n*). MMSE, Mini-Mental State Examination; GDS-15, Geriatric Depression Scale-15 item version; FES-I, Falls Efficacy Scale-International. | | | | | |

**Supplementary Table 3.** Multivariable regression analysis for locomotor capacity at discharge that includes candidate variables identified in the univariable analysis and adjusted for primary diagnosis for admission.

| **Variable** | **Multivariable analysis** | |
| --- | --- | --- |
|  | *ß* | *p* |
| Gender^a^ | −0.578 | 0.129 |
| BMI |  |  |
| Normal (ref.) | - | - |
| Underweight | −0.666 | 0.109 |
| Overweight | −0.227 | 0.636 |
| Frailty | −1.179 | 0.005 |
| Mean daily PA level^b^ | 0.817 | 0.007 |
| SPPB at admission | 0.715 | <0.001 |
| Primary diagnosis for admission |  |  |
| Musculoskeletal (ref.) | - | - |
| Neurological | −0.266 | 0.625 |
| Infectious | −0.178 | 0.777 |
| Cardiovascular | −1.635 | 0.020 |
| Gastrointestinal | −1.224 | 0.102 |
| Neuromusculoskeletal | 0.107 | 0.888 |
| General health deterioration | −0.994 | 0.185 |
| Others | −0.716 | 0.246 |
| Adjusted *R^2^* = 0.612 | | |
| ^a^Female=0, male=1. ^b^*ß* given for an increase of 0.01 METs. BMI, Body Mass Index; PA, physical activity; SPPB, Short Physical Performance Battery. | | |

**Supplementary Table 4.** Multivariable regression analysis for physical activity at discharge that includes candidate variables identified in the univariable analysis and adjusted for primary diagnosis for admission.

| **Variable** | **Multivariable analysis** | |
| --- | --- | --- |
|  | *ß* | *p* |
| Primary locomotion mode |  |  |
| Independent walking (ref.) | - | - |
| Walking with AMD | −0.485 | 0.322 |
| Wheelchair dependent | −2.233 | 0.005 |
| Frailty | −0.664 | 0.035 |
| Barthel Index | 0.021 | 0.018 |
| SPPB | 0.817 | 0.868 |
| Step count at admission^a^ | 0.364 | <0.001 |
| Primary diagnosis for admission |  |  |
| Musculoskeletal (ref.) | - | - |
| Neurological | 0.449 | 0.287 |
| Infectious | 0.344 | 0.469 |
| Cardiovascular | −0.555 | 0.335 |
| Gastrointestinal | −1.364 | 0.011 |
| Neuromusculoskeletal | 0.170 | 0.785 |
| General health deterioration | −0.617 | 0.299 |
| Others | −1.430 | 0.005 |
| Adjusted *R^2^* = 0.563 | | |
| ^a^Natural log-transformed. AMD, assistive mobility device; SPPB, Short Physical Performance Battery. | | |

**Supplementary Table 5.** Multivariable regression analysis for life-space mobility at discharge that includes candidate variables identified in the univariable analysis and adjusted for primary diagnosis for admission.

| **Variable** | **Multivariable analysis** | |
| --- | --- | --- |
|  | *ß* | *p* |
| Cognitive impairment | −2.869 | 0.145 |
| Frailty | −6.302 | 0.005 |
| LSA-IS-T at admission | 0.659 | <0.001 |
| Primary diagnosis for admission |  |  |
| Musculoskeletal (ref.) | - | - |
| Neurological | −2.856 | 0.337 |
| Infectious | −2.313 | 0.465 |
| Cardiovascular | −5.158 | 0.179 |
| Gastrointestinal | −3.947 | 0.274 |
| Neuromusculoskeletal´ | −0.415 | 0.918 |
| General health deterioration | −7.712 | 0.059 |
| Others | −3.336 | 0.265 |
| Adjusted *R^2^* = 0.312 | | |
| LSA-IS-T, Life-Space Assessment in Institutionalized Settings, total score. | | |
